# Editorial: Molecular and Cellular Mechanisms of the Legume-Rhizobia Symbiosis

**DOI:** 10.3389/fpls.2018.01839

**Published:** 2018-12-12

**Authors:** Jianping Wang, Stig Uggerhøj Andersen, Pascal Ratet

**Affiliations:** ^1^Key Laboratory of Genetics, Breeding and Multiple Utilization of Crops, Ministry of Education, Fujian Provincial Key Laboratory of Haixia Applied Plant Systems Biology, Center for Genomics and Biotechnology, Fujian Agriculture and Forestry University, Fuzhou, China; ^2^Agronomy Department, University of Florida, Gainesville, FL, United States; ^3^Department of Molecular Biology and Genetics, Aarhus University, Aarhus, Denmark; ^4^Institute of Plant Sciences Paris-Saclay IPS2, CNRS, INRA, Université Paris-Sud, Université Evry, Université Paris-Saclay, Orsay, France; ^5^Institute of Plant Sciences Paris-Saclay, Paris Diderot, Sorbonne Paris-Cité, Orsay, France

**Keywords:** legume, rhizobium, nodule, symbiosis, SYM pathway, biological nitrogen fixation

Legume-rhizobia symbiosis is a remarkable and mutually beneficial association between higher plants and microbes, which is extremely important for sustainable agriculture and ecology. During this association, biological nitrogen fixation occurs in the nodule, which is a specialized accessory legume organ, generally formed on roots. In mature nodules, rhizobia convert inert atmospheric N_2_ into ammonia (NH_3_), essential for plant growth. In return, bacteria obtain photosynthetic carbon from the plant. The biologically fixed nitrogen during symbiosis accounts for approximately 65% of nitrogen use in agriculture (Burris and Roberts, 1993).

Due to its agricultural importance and interesting biological features, the legume-rhizobia symbiosis fascinated many researchers in the past decades. It has been discovered that the legume-rhizobia symbiosis is initiated through mutual chemical communication. The plant root releases flavonoids, which induce rhizobia to produce specific lipo-chitooligosaccharides (LCOs), called nodulation factors (Nod factors, or NFs) (Dénarié et al., 1996). These NFs are specifically recognized by the host plant and trigger a sophisticated symbiotic signaling cascade in the host root cells to coordinate rhizobial infection, nodule organogenesis and later on nitrogen fixation (Oldroyd et al., 2011). The main aim of this research topic was to assemble papers addressing and discussing the fundamental science of the molecular mechanisms of legume-rhizobia symbiosis. A total of nine articles were published under this research topic related to rhizobium characters (2), plant response to different type of bacteria (1), phytohormones (3), SYM pathway signals (2), and R genes determining specificity of rhizobia infection (1).

Rhizobia have two lifestyles, a free-living state in soil and a symbiotic state in plant cells when engaged in symbiosis (Gibson et al., 2008). Rhizobia therefore have to adapt to changing conditions outside and inside the host plants. Besides LCOs, a few other bacterial components are important for a successful symbiotic interaction. Lipopolysaccharides (LPSs) are major components of the outer membrane of gram negative bacteria including rhizobia and play a central role in rhizobia infection, in adaptation to the host environment, and in facilitating symbiosis (Lerouge and Vanderleyden, 2002; Raetz and Whitfield, 2002). Busset et al. characterized a cluster of five genes presumably involved in LPS biosynthesis and investigated the specific roles of one gene (*lpxXL*) in *Bradyrhizobium* free-living and symbiotic states.

Some rhizobia can also use a type 3 secretion system (T3SS) to translocate bacterial effector proteins into host cells to trigger and facilitate symbiosis in certain legume species (Jiménez-Guerrero et al., 2015; Okazaki et al., 2016). Songwattana et al. examined the role of a T3SS present on a *Bradyrhizobium* symbiotic plasmid. The symbiotic performance of a T3SS mutant *Bradyrhizobium* strain was tested on 9 different legume species. In addition, the role of the R3SS in *Bradyrhizobium* endophytic colonization of rice roots was examined. This work confirmed that the T3SS plays significant roles in symbiosis for certain legume species.

Legume plants, when perceiving compatible NFs, respond very rapidly to rhizobial infection at the physiological and molecular levels (Desbrosses and Stougaard, 2011; Oldroyd, 2013). However, how legumes differentiate compatible from incompatible rhizobia or pathogens was not clear. Kelly et al. investigated the transcriptome profiles of *Lotus japonicus* in response to a spectrum of interacting bacteria, including compatible symbiotic *Mesorhizobium loti*, semi-compatible symbiotic *Sinorhizobium fredii*, incompatible symbiotic *Bradyrhizobium elkanii*, and pathogenic *Pseudomonas syringae* and *Ralstonia solanacearum*. This comparative analysis revealed distinct transcriptional responses of the legume plant to different types of bacteria and challenged the concept that an early defense-like response is evoked by compatible rhizobia in *L. japonicus* during the establishment of symbiosis.

Rhizobial infection and organogenesis events during legume-rhizobia symbiosis are tightly coordinated, and defects in the infection process often result in defective nodule organogenesis. The tight coordination with signaling across root layers strongly suggests the involvement of hormonal pathways. The mini review presented by Buhian and Bensmihen summarized how phytohormones, as key regulators of cellular and developmental plasticity in plants, control the symbiosis process, specifically the early symbiotic events involving NFs. New findings illustrating NF and phytohormone signaling crosstalk in controlling symbiosis establishment were summarized and synergized comprehensively in this mini review.

Cytokinins are essential phytohormones for rhizobia infection and nodule development, particularly for initiation of root cortical cell divisions and induction of critical gene expression in the NF-signaling pathway. To further investigate the role of cytokinin in controlling nodule numbers in soybean. Mens et al. evaluated and characterized the *IPT* gene family involved in cytokinin biosynthesis in soybean with respect to gene number, sequences, and expression in response to nodulation and soil nitrogen. The results illustrated the exact function of various *IPT* genes in soybean nodulation and indicated that in general low levels of cytokinins promote nodulation, whereas high levels inhibit it possibly due to toxic effects, and interestingly *IPT* genes may not play a role in the autoregulation of nodulation pathway.

Two different types of nodules, indeterminate and determinate, are generally formed by legumes. The model legume *Medicago truncatula* forms indeterminate nodules (typically elongate and maintaining an apical nodule meristem), while *L. japonicus* forms determinate nodules (typically round with no meristematic activity in mature nodules (Hirsch, 1992). What determines the difference between these two organogenesis programs is largely unknown. The phytohormone auxin is essential for cell divisions in plants and required for both indeterminate or determinate nodule development (Suzaki et al., 2012). Ng and Mathesius carried out a detailed analysis of auxin transport and localization during indeterminate and determinate nodule formation. Their results suggest that auxin response is observed in pericycle, endodermis, and inner cortex cells of the roots of indeterminate nodule-forming species, while increased auxin responses in outer cortex cells likely take place during determinate nodule formation. Specifically, the tissue difference correlates nicely with the fact that the initial cell divisions start in pericycle and endodermis in *M. truncatula* (indeterminate nodules) while the first cell divisions leading to determinate nodule formation initiate in the outer cortex for plants with determinate nodules.

Research on the legume-rhizobia symbiosis benefits from the advances of technologies at the cellular and molecular levels. Cellular calcium oscillations are critical for signal transduction. The analysis of Ca^2+^ signaling in different cellular compartments requires tools adapted for monitoring Ca^2+^ dynamics with high spatial and temporal resolution. Kelner et al. demonstrated the successful application of dual color sensors for simultaneously monitoring calcium signal dynamics *in vivo* in single root cell compartments during symbiosis, thus providing a powerful tool to study the signal transduction involving Ca^2+^ signaling.

CCaMK is a calcium/calmodulin-dependent protein kinase localized in the nucleus and plays a critical role downstream of symbiotic calcium spiking. Activated by Ca^2+^ and Ca^2+^-loaded calmodulin, CCaMK phosphorylates CYCLOPS or IPD3 in *M. truncatula* (Yano et al., 2008). Jauregui et al. demonstrated that mutations in the calmodulin-binding domain of CCaMK alter its calmodulin binding capacity, generating a constitutively activated kinase that confers an altered nodulation phenotype.

Particular rhizobial species or strains normally nodulate only a narrow group of legume species or genotypes (Wang et al., 2012, 2017). Understanding the genetic and molecular basis of this symbiotic specificity is important for developing strategies to improve symbiosis. Through genetic mapping Fan et al. identified a dominant gene in soybeans that restricts nodulation with *S. fredii* USDA193. Further validation indicated that an *Rfg1* allele encoding a member of the Toll-interleukin receptor/nucleotide-binding site/leucine-rich repeat class of plant resistance proteins restricts nodulation by *S. fredii* strains USDA257 and USDA205. In combination with several previous studies, they showed that the *Rfg1* allele likely provided broad-spectrum resistance to nodulation by many *S. fredii* and *B. japonicum* strains in soybean.

## Author Contributions

JW prepared the first draft of the manuscript. SA and PR revised the draft critically and constructively. All authors reviewed and approved the final draft.

### Conflict of Interest Statement

The authors declare that the research was conducted in the absence of any commercial or financial relationships that could be construed as a potential conflict of interest.
